# Hexaraphane Affects the Activation of Hepatic PPARα Signaling: Impact on Plasma Triglyceride Levels and Hepatic Senescence with Aging

**DOI:** 10.3390/nu17111768

**Published:** 2025-05-23

**Authors:** Manami Higa, Kazuma Naito, Takenari Sato, Ayame Tomii, Yuuka Hitsuda, Miyu Tahara, Katsunori Ishii, Yu Ichisaka, Hikaru Sugiyama, Rin Kobayashi, Fuzuki Sakamoto, Kazuhisa Watanabe, Keisuke Yoshikiyo, Hidehisa Shimizu

**Affiliations:** 1 Graduate School of Natural Science and Technology, Shimane University, 1060 Nishikawatsu-Cho, Matsue 690-8504, Shimane, Japan; 2 The United Graduate School of Agricultural Sciences, Tottori University, 4-101 Koyama-Minami, Tottori 680-8553, Tottori, Japan; 3 Faculty of Life and Environmental Sciences, Shimane University, 1060 Nishikawatsu-Cho, Matsue 690-8504, Shimane, Japan; 4 Faculty of Nutrition, Tokyo Kasei University, 1-18-1 Kaga, Itabashi 173-8602, Tokyo, Japan; 5 Institute of Agricultural and Life Sciences, Academic Assembly, Shimane University, 1060 Nishikawatsu-Cho, Matsue 690-8504, Shimane, Japan; 6 Estuary Research Center, Shimane University, 1060 Nishikawatsu-Cho, Matsue 690-8504, Shimane, Japan; 7 Interdisciplinary Center for Science Research, Shimane University, 1060 Nishikawatsu-Cho, Matsue 690-8504, Shimane, Japan

**Keywords:** 6-MSITC, PPARα, CPT1A, ACOX1, Sirt1, p21, plasma triglyceride level, hepatic senescence, aging

## Abstract

**Background/Objectives**: Hexaraphane, also known as 6-methylsulfinylhexyl isothiocyanate, derived from wasabi (*Eutrema japonicum*), increases heme oxygenase-1 (HO-1) and aldehyde dehydrogenase 2 (ALDH2) mRNA expression by activating nuclear factor erythroid 2-related factor 2 (Nrf2) in both HepG2 cells and the mouse liver. Given the presence of a peroxisome proliferator-activated receptor (PPAR) response element (PPRE) in the HO-1 and ALDH2 promoters, the present study aimed to determine the effects of hexaraphane on PPARα-associated genes, age-related weight gain, plasma triglyceride levels, and hepatic senescence. **Methods**: HepG2 cells were treated with hexaraphane to evaluate PPARα target gene expression and PPRE transcriptional activity. Male C57BL/6J young control, aged control, and aged mice administered with hexaraphane for 16 weeks were assessed for food and water intake, body and tissue weights, plasma parameters, and hepatic PPARα-related gene expression. **Results**: Hexaraphane increased HO-1 mRNA expression levels in HepG2 cells, which was inhibited by GW6471, a PPARα antagonist. It elevated PPRE transcriptional activity and increased carnitine palmitoyltransferase 1A (CPT1A) mRNA expression levels, indicating PPARα activation. In aged mice, hexaraphane intake reduced body weight gain by decreasing the adipose tissue weight. Increased CPT1A expression levels and a tendency toward increased acyl-CoA oxidase 1 (ACOX1) expression levels in the liver of aged mice administered hexaraphane were associated with reduced plasma triglyceride levels and body weight gain. Increased hepatic Sirt1 expression levels in aged mice administered hexaraphane was associated with lower plasma triglyceride levels. Increased hepatic PPARα mRNA expression levels in aged mice administered hexaraphane suggest a positive feedback loop between PPARα and Sirt1. The expression levels of hepatic p21 mRNA, a senescence marker regulated by Sirt1, were upregulated in aged mice but suppressed by hexaraphane intake. **Conclusions**: Hexaraphane may prevent age-related body weight gain, elevated plasma triglyceride levels, and hepatic senescence by activating PPARα, potentially contributing to longevity.

## 1. Introduction

Recent advancements in medical science have markedly increased global life expectancy. Projections suggest that by 2050, the population aged ≥ 60 years will surpass two billion, largely because of these advancements [1]. As people age, the prevalence of numerous age-related diseases increases [2,3,4]. In particular, both the incidence of liver disease and its associated mortality rates increase with age [4,5]. Therefore, it is crucial to address the decline in liver function and the progression of hepatic senescence that accompany aging, as these factors contribute to the development of liver disease. This approach is essential not only for enhancing life expectancy but also for extending the healthspan of the elderly.

Impaired energy metabolism is a common feature of aging [6,7,8]. Because plasma triglyceride levels increase with impaired energy metabolism due to aging [9,10,11,12], they have been proposed as potential biomarkers of aging [11,12]. Furthermore, elevated serum triglyceride levels are associated with premature aging [9]. Triglyceride levels are regulated by peroxisome proliferator-activated receptor α (PPARα), which is primarily expressed in the metabolically active liver [13,14]. Activation of PPARα enhances fatty acid β-oxidation (FAO) activity by upregulating the expression of rate-limiting enzymes, such as carnitine palmitoyltransferase 1A (CPT1A) and acyl-CoA oxidase 1 (ACOX1), which are localized in the mitochondria and peroxisomes, respectively. This process reduces the availability of fatty acids for triglyceride storage, resulting in decreased triglyceride levels [8,9]. Therefore, the increased expression of FAO-related genes, such as CPT1A and ACOX1, induced by PPARα activation, is expected to ameliorate impaired energy metabolism, leading to lower plasma triglyceride levels and potentially delayed aging. In fact, various non-neoplastic spontaneous aging lesions occur with a higher incidence, shorter latency, and more severe symptoms in PPARα knockout mice than in wild-type mice [15]. Furthermore, in addition to PPARα inducing the upregulation of the longevity- and anti-aging-related gene Sirt1 in the liver [16], PPARα knockout mice exhibit a reduced lifespan compared to wild-type mice [15].

Wasabi (*Eutrema japonicum*) is a cruciferous vegetable and traditional Japanese spice. Its rhizomes are widely found in Japan and are extensively used in the Japanese cuisine. The bioactive components of wasabi are isothiocyanate analogs, with hexaraphane (also known as 6-methylsulfinylhexyl isothiocyanate) being the primary bioactive compound [17]. A study involving healthy Japanese individuals aged 60–80 years demonstrated that daily administration of hexaraphane for 12 weeks significantly improved working and episodic memory compared to that in the placebo group [18]. Additionally, owing to its low toxicity risk compared to synthetic compounds, it shows promise for preventing and treating lifestyle-related diseases [19]. Regarding the action mechanism of hexaraphane, it is known to activate the nuclear factor erythroid 2-related factor 2 (Nrf2)/antioxidant response element (ARE) pathway [20] and induces the expression of heme oxygenase-1 (HO-1) and aldehyde dehydrogenase 2 (ALDH2) by activating Nrf2 in the liver [21]. However, since both ARE and PPAR response elements (PPRE) are present in the promoter regions of HO-1 [22,23] and ALDH2 [24,25], we predicted that hexaraphane could activate PPARα. Therefore, the present study aimed to analyze the potential involvement of hexaraphane in PPARα activation in HepG2 cells, using the expression levels of PPARα target genes and PPRE transcriptional activity as indicators. In addition, because the aging process and characteristics of aging rodents (mice aged 12 months or older) are similar to those of humans [26], the present study focused on whether hexaraphane administration to aged mice induces PPARα target gene expression in the liver, thereby affecting plasma triglyceride levels and hepatic senescence, which are associated with age-related diseases.

## 2. Materials and Methods

### 2.1. Materials

Aprotinin, penicillin–streptomycin solution (×100), Dulbecco’s modified Eagle’s medium (DMEM) with low glucose, and dimethyl sulfoxide (DMSO) were purchased from FUJIFILM Wako Pure Chemical Corporation (Osaka, Japan). Heparin was purchased from Nacalai Tesque, Inc. (Kyoto, Japan). Fetal bovine serum (FBS) was obtained from Biowest S.A.S. (Nuaillé, France). GW6471 (a PPARα antagonist) was purchased from Selleck Chemicals (Houston, TX, USA). Hexaraphane for cell culture experiments was obtained from Cayman Chemical (Ann Arbor, MI, USA). Hexaraphane for animal experiments was provided by KINJIRUSHI Co., Ltd. (Nagoya, Japan).

### 2.2. Cell Culture

HepG2 cells, a human hepatocellular carcinoma cell line, were acquired for the present study from the RIKEN Cell Bank (Tsukuba, Japan) and cultured in DMEM supplemented with low glucose, 10% FBS, 100 U/mL penicillin, and 100 g/mL streptomycin at 37 °C in a humidified atmosphere of 95% (*v*/*v*) air and 5% (*v*/*v*) CO_2_. The cells were subsequently incubated in serum-free DMEM for 24 h prior to the experiments. For hexaraphane treatment, Dulbecco’s phosphate-buffered saline (−) (DPBS (−)) was used as both the control and vehicle. For GW6471 and fenofibrate treatments, DMSO was used as the control and vehicle, respectively. The final concentrations of DPBS (−) and DMSO used were 0.1% (*v*/*v*) each.

### 2.3. Animal Experiments

The Animal Care and Use Committee of Shimane University approved all animal experiments and procedures (protocol codes: MA4-03-00; date of approval: August 19, 2022), which were conducted in accordance with the Institutional Regulations of Shimane University and complied with the Act on Welfare and Management of Animals (Act No. 105), and relevant standards and guidelines in Japan. Male wild-type C57BL/6J mice, aged 5 and 68 weeks, were obtained from Jackson Laboratory Japan, Inc. (Yokohama, Japan). The number of animals per cage was limited to two, and the plastic cages were furnished with paper floor coverings to provide an environment conducive to nesting and to minimize the occurrence of aggressive behavior among the mice. The floor coverings were replaced at weekly intervals to minimize environmental stress to the mice. The mice were maintained at 22 ± 2 °C and 55 ± 5% relative humidity in an air-conditioned room with an automated light-dark cycle (light phase: 08:00–20:00). During a 5-day acclimatization period, the mice were provided *ad libitum* access to the CMF diet (Oriental Yeast Co., Ltd., Tokyo, Japan; crude protein 28.1%, crude fat 8.3%, crude ash 6.3%, crude fiber 2.9%, nitrogen-free extract 46.2%, and moisture 8.1%) designed to support the long-term maintenance of mice such as C57BL and water. Following acclimation, the mice were allocated to three groups: young control group (*n* = 10), aged control group (*n* = 10), and aged mice administered hexaraphane (aged hexaraphane group) (*n* = 8). The mean body weights of the two groups of aged mice were adjusted to ensure similarity. Mice were provided *ad libitum* access to the CMF diet and water (young and aged control groups) or hexaraphane (100 µM) dissolved in water for 16 weeks. The humane endpoint was established using age-related illness or distress and excessive weight loss due to hexaraphane consumption as indicators. Throughout the rearing period, the mice were monitored daily for symptoms, with particular emphasis placed on aged mice, which may exhibit abnormalities as they age. Furthermore, to minimize stress on the mice, their food and drinking water or water containing hexaraphane were replenished every 2–3 days, during which time their body weight, food intake, and water intake were measured. To minimize potential confounding variables, the plastic cages used for rearing each group were arranged randomly, and all measurements and dissections conducted during the rearing period were similarly randomized. At the end of the experimental period, the following procedure was used to treat the mice with isoflurane for analgesia, and blood was collected before euthanizing them. Blood was collected from the abdominal vena cava of the mice under anesthesia with 3% isoflurane for induction and 2% isoflurane via nose cone for maintenance using a syringe containing heparin (final concentration 50 U/mL blood) and aprotinin (final concentration 500 kIU/mL blood), and the plasma was subsequently prepared. The abdominal aorta and vena cava were then severed, and the mice were euthanized by exsanguination. The liver, epididymal fat, and gastrocnemius muscle were promptly excised, weighed, rapidly frozen in liquid nitrogen, and stored at −80 °C until analysis.

### 2.4. Quantitative Real-Time PCR

Serum-starved HepG2 cells were incubated with control solution (0.1% DMSO) or GW6471 (10 μM) for 1 h and then stimulated with control solution (0.1% DPBS (−)) or various concentrations of hexaraphane for the indicated periods. Total RNA was extracted from HepG2 cells and mouse livers using Sepasol-RNA I Super G (Nacalai Tesque Corporation, Kyoto, Japan) and RNeasy Mini Kits (QIAGEN, Hilden, Germany), respectively. The mRNA expression levels of target genes from the extracted total RNA were assessed using PrimeScript™ RT Master Mix (Perfect Real Time), TB Green™ Premix Ex Taq™ II (Tli RNaseH Plus), and Thermal Cycler Dice Real Time System III (Takara Bio Inc., Shiga, Japan). These procedures were performed according to our established protocols [27,28]. The oligonucleotide primers used in the present study are listed in Table 1. Amplicons were quantified based on a calibration curve of known DNA concentrations, and quantification cycle (Cq) values were plotted against logarithmic sample concentrations. The mRNA expression levels of each gene were assessed by comparing them to the mRNA of the ribosomal protein lateral stalk subunit P0 (RPLP0, encoded by *RPLP0* [human] and *Rplp0* [mouse]) used as an internal standard. This selection was based on a study that evaluated four common housekeeping genes (RPLP0, β-actin, glycerol-3-phosphate dehydrogenase, and cyclophilin), in which RPLP0 was identified as the most reliable standard for gene expression analysis [29].

### 2.5. Measurement of Cell Viability

Serum-starved HepG2 cells were exposed to either control solution (0.1% DMSO or 0.1% DPBS (−)), hexaraphane (0.5 μM), or GW6471 (10 μM) for 48 h. The extent of cell viability was subsequently evaluated using the Cell Counting Kit-8 (Dojindo, Kumamoto, Japan), following previously established protocols [30,31].

### 2.6. Transfection and Luciferase Assays

PPRE transcriptional activity was measured in HepG2 cells transfected with PPRE X3-TK-luc (Addgene, Cambridge, MA, USA) and pRL-SV40 plasmids (Promega, Madison, WI, USA) using FuGENE HD (Roche, Mannheim, Germany). Following serum starvation for 24 h, transfected HepG2 cells were stimulated with control solution (0.1% DMSO and 0.1% DPBS (−)), hexaraphane (0.5 μM), or fenofibrate (50 μM) for 6 h. Subsequently, firefly luciferase activity was determined and normalized to renilla luciferase activity, which was measured utilizing the Dual-Luciferase Reporter Assay System (Promega, Madison, WI, USA) with a Junior LB9509 luminometer (Berthold Technologies, Bad Wildbad, Germany), adhering to previously established protocols [32]. Values represent the mean of three independent experimental replicates.

### 2.7. Measurements of Plasma Triglyceride

Plasma was prepared by centrifuging the collected blood at 2000× *g* for 10 min at 4 °C. Plasma albumin and triglyceride levels were measured by Oriental Yeast Co., Ltd. (Tokyo, Japan).

### 2.8. Statistical Analysis

Results are expressed as the mean ± standard error (SE). For cell culture experiments, the data were analyzed using Student’s *t*-test. For animal experiments, comparisons between the young control and aged groups were performed using ANOVA with Dunnett’s test. Comparisons between the aged control and aged hexaraphane groups were performed using Student’s *t*-test. Correlations were analyzed using Pearson’s correlation and simple regression analyses. Statistical analyses were performed using Microsoft Excel 2011 (Microsoft Corp., Redmond, WA, USA) and Statcel 4 (OMS Publishing Co., Saitama, Japan). Statistical significance was set at *p* < 0.05.

## 3. Results

### 3.1. Involvement of Hexaraphane in the Activation of PPARα and Increase in the Expression of Its Target Genes

**Figure 1:**
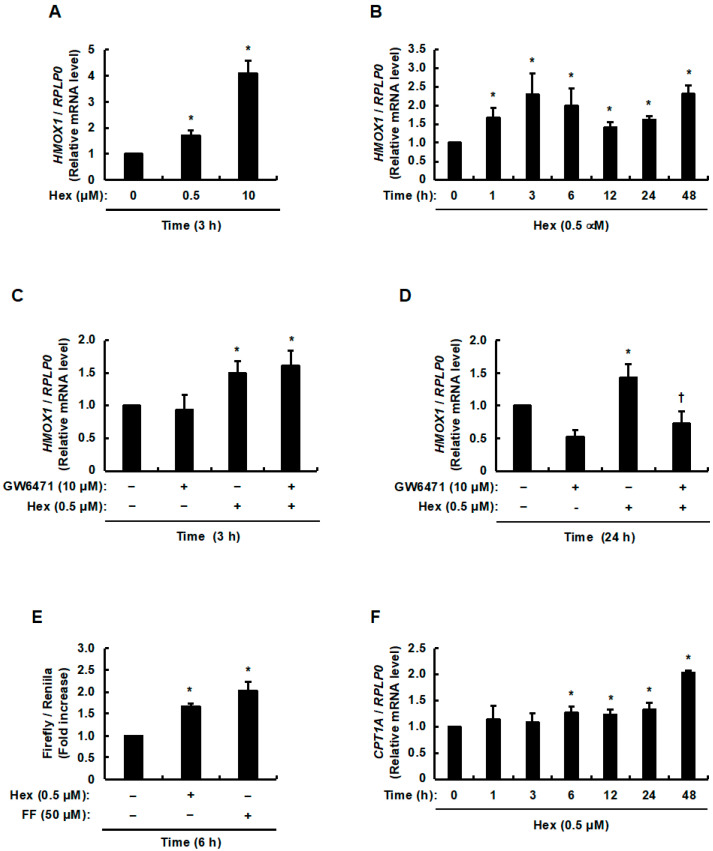
Effects of hexaraphane on the regulation of PPARα target gene expression in HepG2 cells. (**A**) Dose-dependent *HMOX1* mRNA expression levels. (**B**) Time-dependent of *HMOX1* mRNA expression levels. (**C**) Effect of GW6471 on *HMOX1* mRNA expression levels (**C**) 3 and (**D**) 24 h after stimulation with hexaraphane. (**E**) PPRE transcriptional activity. (**F**) Time-dependent of *CPT1A* mRNA expression levels. (**A**,**C**,**E**,**F**) represent the mean ± SE of three independent experiments, and (**B**,**D**) represent the mean ± SE of four independent experiments. * *p* < 0.05 vs. 0 µM, 0 h, control solution (DPBS (−)), or control solution (DMSO). ^†^ *p* < 0.05, vs. hexaraphane alone. Hex, hexaraphane; FF, fenofibrate.

Hexaraphane has been reported to significantly increase HO-1 expression in hepatocytes at a concentration of 10 μM [21]. However, we predicted that hexaraphane would upregulate the mRNA expression levels of human HO-1, encoded by *HMOX1*, at concentrations below 10 µM. As expected, stimulation of HepG2 cells with hexaraphane increased *HMOX1* mRNA expression levels not only at 10 µM but also at 0.5 µM (Figure 1A). We examined the time course from 1 to 48 h after stimulation with 0.5 µM hexaraphane and observed peak *HMOX1* mRNA expression levels at 3 and 48 h post-stimulation (Figure 1B). Given the presence of PPRE in the promoter region of *HMOX1* [22,23], we sought to determine whether hexaraphane is involved in the upregulation of HO-1 expression via PPARα activation. Pretreatment with GW6471, a PPARα antagonist, did not affect the increase in *HMOX1* mRNA expression levels induced by hexaraphane after 3 h (Figure 1C). Considering that another peak in *HMOX1* mRNA expression levels occurred 48 h after stimulation, we conducted a preliminary assessment of the cell viability of hexaraphane and GW6471 on HepG2 cells after 48 h of treatment. Exposure of HepG2 cells to hexaraphane during this period did not affect their viability (Supplementary Figure S1). In contrast, treatment of HepG2 cells with GW6471 for the same duration resulted in decreased cell viability (Supplementary Figure S2). Based on this result, when HepG2 cells pretreated with GW6471 were stimulated with 0.5 µM hexaraphane for 24 h instead of 48 h, a decrease in the hexaraphane-induced increase in *HMOX1* mRNA expression levels was observed (Figure 1D). As these results suggested that hexaraphane is involved in PPARα activation, we examined whether hexaraphane elevated the transcriptional activity of PPRE. A luciferase assay using the PPRE response sequence showed that hexaraphane, as well as fenofibrate, elevated PPRE transcriptional activity (Figure 1E). In addition, the mRNA expression levels of human CPT1A, encoded by *CPT1A*, a typical PPARα target gene, was examined. We found that hexaraphane dramatically and significantly increased *CPT1A* mRNA expression levels 48 h after stimulation (Figure 1F). As mentioned above, because cell death was induced by GW6471 treatment for 48 h (Supplementary Figure S2), it was difficult to use GW6471 to confirm whether PPARα activation was involved in the hexaraphane-induced increase in *CPT1A* mRNA expression levels. Nevertheless, these findings suggest that hexaraphane may function as a PPARα activator, and that PPARα activation by hexaraphane induces increased mRNA expression levels of both HO-1 and CPT1A.

### 3.2. Oral Administration of Hexaraphane Affects the Body Weight in Aged Mice

**Figure 2:**
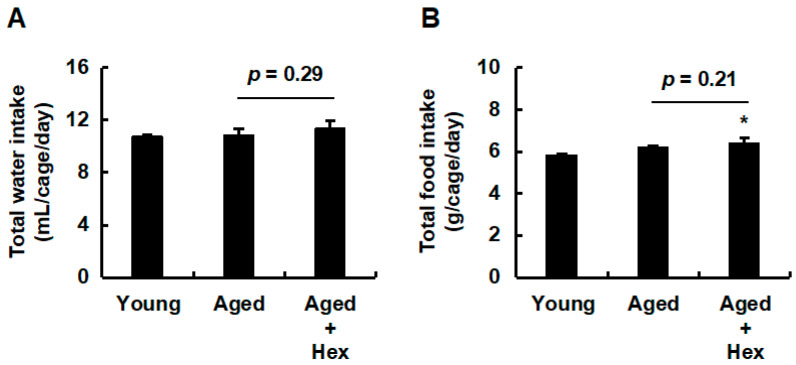
Total water and food intake. (**A**) Water intake. (**B**) Food intake. Data are presented as the mean ± SE for each mouse group. * *p* < 0.05, vs. young control group. Aged, aged control group; Aged + Hex, aged mice administered hexaraphane group; Young, young control group.

**Figure 3:**
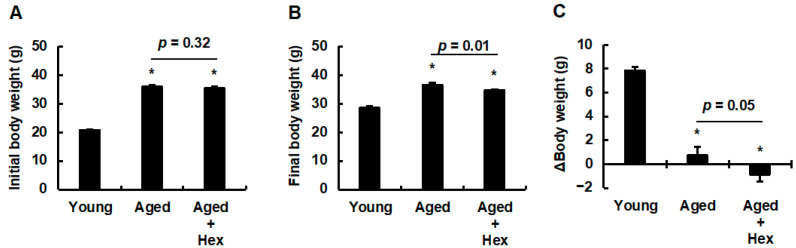
Initial and final body weights and Δbody weights. (**A**) Initial and (**B**) final body weights. (**C**) Δbody weights. Data are presented as the mean ± SE for each mouse group. * *p* < 0.05, vs. young control group. Aged, aged control group; Aged + Hex, aged mice administered hexaraphane group; Young, young control group.

Given the promising in vitro effects of hexaraphane on PPARα activation, the present study examined its effects in aged mice. Young mice were used as controls to confirm the progression of aging. The hexaraphane concentration in mouse drinking water was determined based on a study using *App* knock-in Alzheimer’s disease model mice administered water containing 0.4 mg/mL hexaraphane [33]. In the present study, as HepG2 cells treated with 0.5 μM hexaraphane (1/20 of 10 μM) exhibited increased HO-1 expression, aged mice received 100 μM hexaraphane dissolved in water (approximately 0.02 mg/mL, 1/20 of 0.4 mg/mL) *ad libitum* for 16 weeks. Figure 2A shows that no significant difference was observed in the water intake among the young control, aged control, and aged hexaraphane groups. Regarding food intake, the aged hexaraphane group consumed significantly more food than the young control group; however, no significant difference was observed between the aged control and aged hexaraphane groups (Figure 2B). Naturally aged mice have been reported to exhibit large individual variations [34]. Therefore, we performed a box-plot outlier analysis to identify outliers using body weight as a health indicator (Supplementary Figure S3). After excluding outliers, we analyzed body weight and Δbody weight. Figure 3A shows that the initial body weights of both the aged control and aged hexaraphane groups were significantly higher than those of the young control group. In addition, the initial body weights of the aged control and aged hexaraphane groups were similar. Both groups of aged mice had significantly higher final body weights than the young control group (Figure 3B). However, the final body weight of the aged hexaraphane group was significantly lower than that of the aged control group (Figure 3B). Analysis of Δbody weight, which quantifies the disparity between final and initial body weights, revealed a marked increase in the young control group (Figure 3C). In addition, the aged control group exhibited only modest increases in body weight (Figure 3C). However, no such increase was observed in the aged hexaraphane group (Figure 3C). When comparing the Δbody weight of the aged control and aged hexaraphane groups, a tendency for suppressed weight gain was observed in the aged hexaraphane group compared to that in the aged control group (*p* = 0.05) (Figure 3C). These results suggest that hexaraphane attenuates age-associated body weight gain.

### 3.3. Oral Administration of Hexaraphane Affects Adipose Tissue Weight but Not Skeletal Muscle Weight in Aged Mice

**Figure 4:**
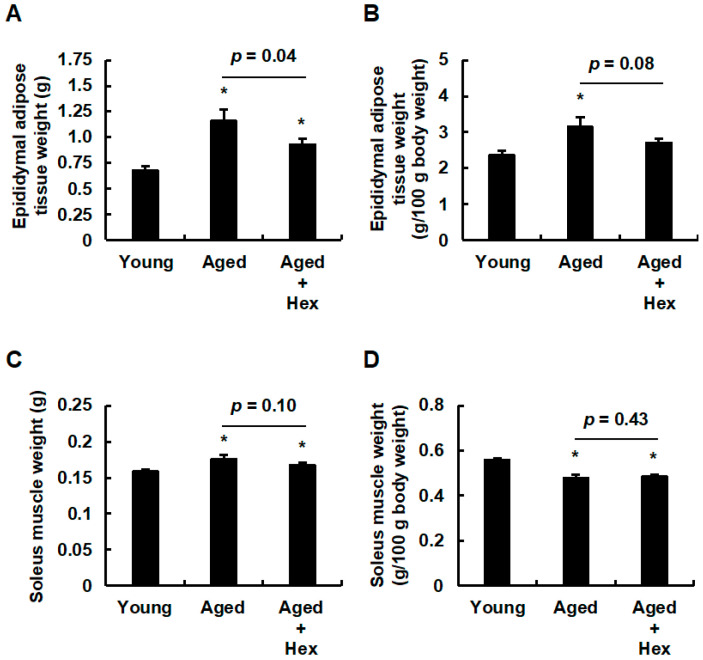
Adipose tissue and skeletal muscle weights. (**A**) Epididymal adipose tissue weights. (**B**) Epididymal adipose tissue weight relative to body weight. (**C**) Gastrocnemius muscle weights. (**D**) Gastrocnemius muscle weight relative to body weight. Data are presented as the mean ± SE for each mouse group. * *p* < 0.05, vs. young control group. Aged, aged control group; Aged + Hex, aged mice administered hexaraphane group; Young, young control group.

To elucidate the factors contributing to the lower body weight and the tendency to suppress body weight gain in the aged hexaraphane group, we measured the weight of the epididymal adipose tissue, a type of adipose tissue, and gastrocnemius muscle, a type of skeletal muscle. Epididymal adipose tissue weight was significantly greater in both the aged control and aged hexaraphane groups than in the young control group (Figure 4A). However, it was lower in the aged hexaraphane group than in the aged control group (Figure 4A). The epididymal fat weight relative to body weight was significantly higher in the aged control group than in the young control group, with no significant difference between the young control and aged hexaraphane groups (Figure 4B). In the comparison between the aged control and aged hexaraphane groups, the epididymal fat weight per body weight exhibited a tendency to decrease in the aged hexaraphane group (*p* = 0.08) (Figure 4B). The gastrocnemius muscle weight was significantly greater in both aged groups than in the young control group (Figure 4C), and the aged hexaraphane group exhibited a tendency toward lower weight than the aged control group (*p* = 0.10) (Figure 4C). The gastrocnemius muscle weight relative to body weight was significantly lower in both aged groups than in the young control group (Figure 4D). In the comparison between the aged control and the aged hexaraphane groups, no significant difference was observed. (Figure 4D). Based on these data, a comprehensive comparison of the data for adipose tissue and skeletal muscle weights in the aged control and aged hexaraphane groups indicated that the observed weight loss in aged mice administered hexaraphane was attributable to a reduction in adipose tissue weight rather than a decrease in skeletal muscle weight.

### 3.4. Oral Administration of Hexaraphane Affects Plasma Triglyceride Levels but Not Plasma Albumin Levels in Aged Mice

**Figure 5:**
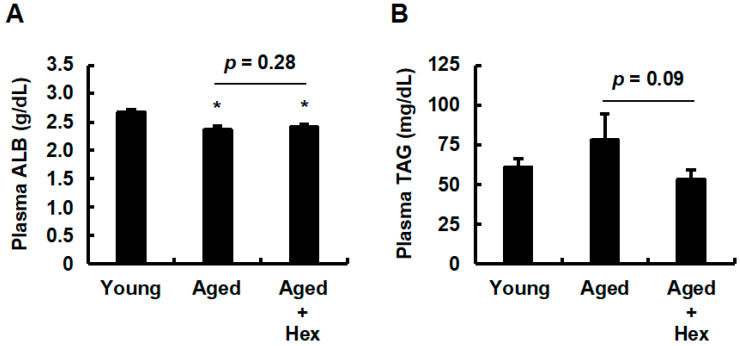
Plasma albumin and triglyceride levels. (**A**) Plasma albumin levels. (**B**) Plasma triglyceride levels. Data are presented as the mean ± SE for each mouse group. * *p* < 0.05, vs. young control group. Aged, aged control group; Aged + Hex, aged mice administered hexaraphane group; TAG, triglyceride; Young, young control group.

Plasma albumin levels are known to be lower in the elderly than in the young due to age-related liver dysfunction, which reduces the protein synthesis capacity [35,36,37,38]. This phenomenon has also been observed in D-galactose-induced aging mice [38,39]. To determine whether hexaraphane supplementation can counteract the age-related decline in protein synthesis, we measured plasma albumin levels. Our findings demonstrated a significant reduction in plasma albumin levels in both aged groups compared to the young control group, with no significant difference between the aged control and aged hexaraphane groups (Figure 5A). In addition, plasma triglyceride levels, which have been proposed as potential biomarkers of aging [11,12], were also measured. These levels tended to be elevated in the aged control group compared to the young control group and decreased in the aged hexaraphane group compared to those in the aged control group (Figure 5B). This indicates that hexaraphane supplementation may attenuate age-related increases in plasma triglyceride levels. These results suggest that while hexaraphane supplementation did not improve the diminished capacity for protein synthesis caused by aging, it alleviated the elevated plasma triglyceride levels associated with aging.

### 3.5. The Relationship Between Hepatic CPT1A Expression Levels and Plasma Triglyceride Levels or Body Weight Gain in Aged Mice Administered Hexaraphane

**Figure 6:**
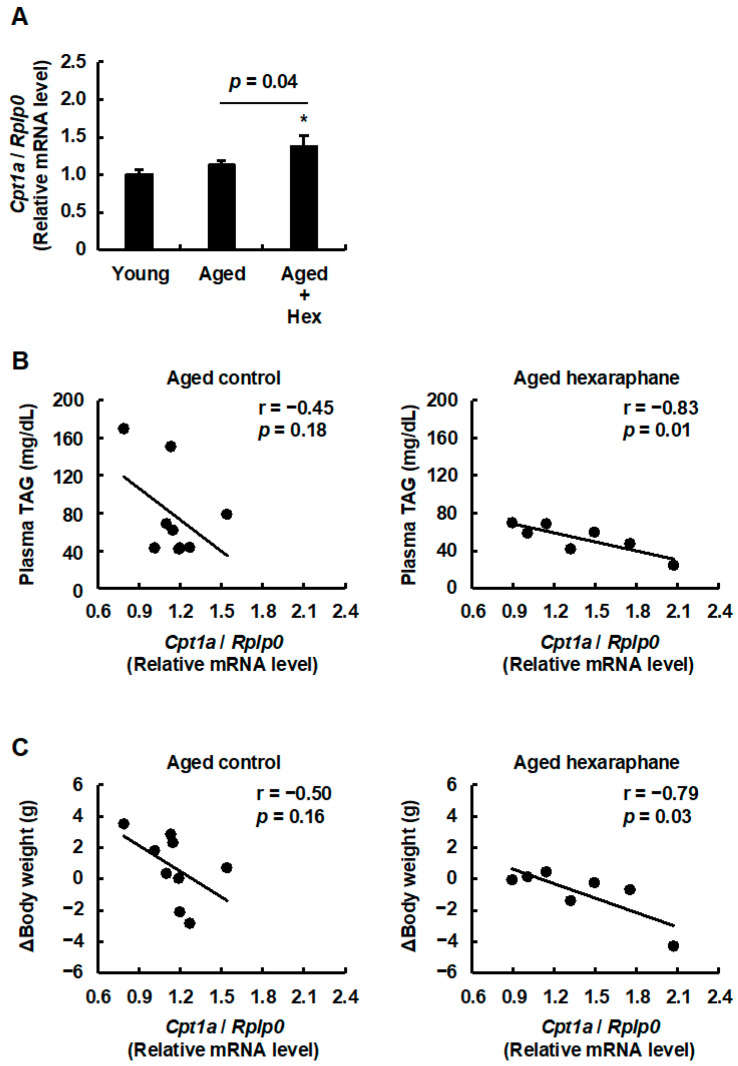
Effects of hepatic CPT1A mRNA expression on plasma triglyceride levels and Δbody weight. (**A**) *Cpt1a* mRNA expression levels. (**B**) Correlation between hepatic *Cpt1a* mRNA expression and plasma triglyceride levels. (**C**) Correlation between hepatic *Cpt1a* mRNA expression levels and Δbody weight. Data are presented as the mean ± SE for each mouse group in (**A**). * *p* < 0.05, vs. young control group for (**A**). The values are presented for each mouse group in (**B**,**C**). Aged, aged control group; Aged control, aged control group; Aged + Hex, aged mice administered hexaraphane group; Aged hexaraphane, aged mice administered hexaraphane group; TAG, triglyceride; Young, young control group.

We examined whether the mRNA expression levels of mouse CPT1A, encoded by *Cpt1a*, a target gene of PPARα and a rate-limiting enzyme for mitochondrial FAO, were upregulated in the livers of aged mice administered hexaraphane, as observed in the results obtained with HepG2 cells. Figure 6A shows that *Cpt1a* mRNA expression levels were significantly higher in the aged hexaraphane group than in the young and aged control groups. Given that increased CPT1A expression enhances mitochondrial FAO metabolism, we predicted that hexaraphane intake would attenuate the elevation of plasma triglyceride levels and Δbody weight. In accordance with this prediction, regression analysis of *Cpt1a* mRNA expression and plasma triglyceride levels revealed a statistically significant negative correlation in the aged hexaraphane group but not in the aged control group (Figure 6B). In addition, a significant negative correlation was observed between *Cpt1a* mRNA expression levels and Δbody weight in the aged hexaraphane group but not in the aged control group (Figure 6C). These findings suggest that hexaraphane functions as a PPARα activator in the livers of aged mice and contributes to reduced plasma triglyceride levels and body weight gain by increasing CPT1A expression levels.

### 3.6. The Relationship Between Hepatic ACOX1 Expression Levels and Plasma Triglyceride Levels or Body Weight Gain in Aged Mice Administered Hexaraphane

**Figure 7:**
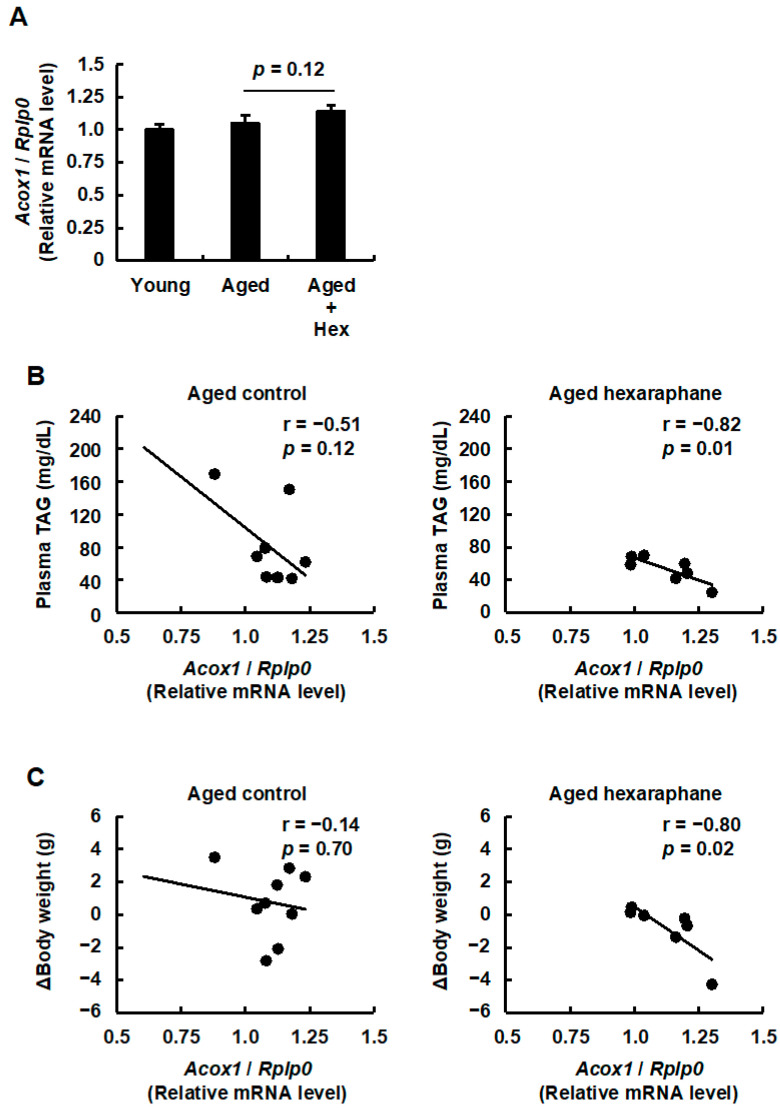
Effects of hepatic ACOX1 mRNA expression on plasma triglyceride levels and Δbody weight. (**A**) *Acox1* mRNA expression levels. (**B**) Correlation between hepatic *Acox1* mRNA expression and plasma triglyceride levels. (**C**) Correlation between hepatic *Acox1* mRNA expression levels and Δbody weight. Data are presented as the mean ± SE for each mouse group in (**A**). * *p* < 0.05, vs. young control group for (**A**). The values are presented for each mouse group in (**B**,**C**). Aged, aged control group; Aged control, aged control group; Aged + Hex, aged mice administered hexaraphane group; Aged hexaraphane, aged mice administered hexaraphane group; TAG, triglyceride; Young, young control group.

We also evaluated the mRNA expression levels of mouse ACOX1, encoded by *Acox1*, which is a target gene of PPARα and a rate-limiting enzyme for peroxisomal FAO, in the liver. As shown in Figure 7A, there were no statistically significant differences in *Acox1* mRNA expression levels among the three groups. However, *Acox1* mRNA expression levels tended to increase in the aged hexaraphane group compared to those in the aged control group. Furthermore, regression analysis of plasma triglyceride levels and Δbody weight against *Acox1* mRNA expression levels revealed statistically significant negative correlations in the aged hexaraphane group, whereas no such correlations were observed in the aged control group (Figure 7B,C). These findings suggest that hexaraphane may aid in reducing plasma triglyceride levels and body weight gain in aged mice through peroxisomal FAO, although its effect may be less pronounced than that of mitochondrial FAO, mediated by CPT1A.

### 3.7. The Relationship Between Hepatic Sirt1 Expression Levels and Plasma Triglyceride Levels, Body Weight Gain, PPARα Expression, or Hepatic Senescence in Aged Mice Administered Hexaraphane

**Figure 8:**
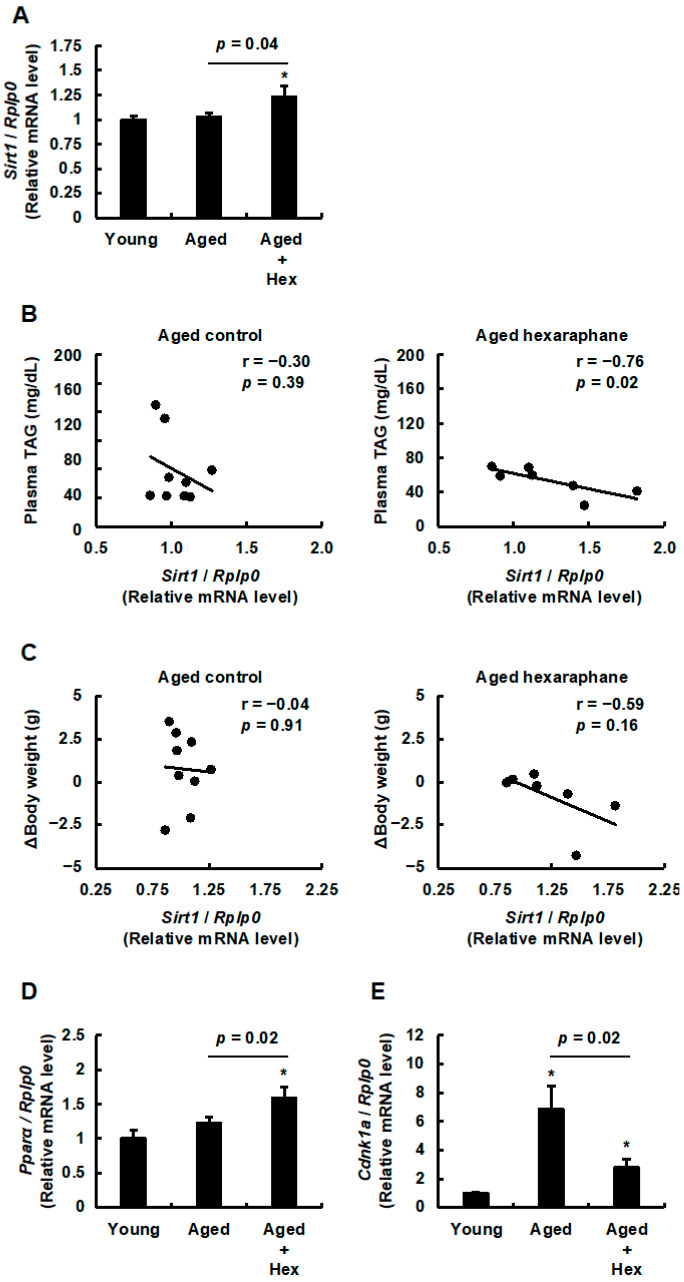
Effects of hepatic Sirt1 mRNA expression on plasma triglyceride concentration, changes in body weight, and mRNA expression of hepatic Sirt1-related genes. (**A**) Sirt1 mRNA expression levels. (**B**) Correlation between hepatic Sirt1 mRNA expression and plasma triglyceride levels. (**C**) Correlation between hepatic Sirt1 mRNA expression levels and Δbody weight. (**D**) PPARα mRNA expression levels. (**E**) p21 mRNA expression levels. Data are presented as the mean ± SE for each mouse group in (**A**,**D**,**E**). * *p* < 0.05, vs. young control group in (**A**,**D**,**E**). Values are presented for each mouse group in (**B**,**C**). Aged, aged control group; Aged control, aged control group; Aged + Hex, aged mice administered hexaraphane group; Aged hexaraphane, aged mice administered hexaraphane group; TAG, triglyceride; Young, young control group.

Sirt1 has a PPRE sequence in its promoter [16], and increased Sirt1 expression is observed in mice livers upon PPARα activation [16]. Based on these reports, we examined whether hexaraphane intake increases the mRNA expression levels of mouse Sirt1, encoded by *Sirt1*, in the livers of aged mice. Figure 8A shows a significant increase in *Sirt1* mRNA expression levels in the aged hexaraphane group compared to those in both the young and aged control groups. In addition, a significant negative correlation was found between *Sirt1* mRNA expression levels and plasma triglyceride levels in the aged hexaraphane group, but not in the aged control group (Figure 8B). Regarding Δbody weight, no significant correlation was found in either the aged control or aged hexaraphane groups (Figure 8C). However, a significant negative correlation was observed when the outliers were removed from the aged hexaraphane group (Supplementary Figure S4). As PPARα and Sirt1 form a positive feedback loop [16,40], we assessed the mRNA expression levels of mouse PPARα, encoded by *Ppara*, and found that they were significantly higher in the aged hexaraphane group than in the young and aged control groups (Figure 8D). Sirt1 is a gene known for its role in preventing senescence across various organs and tissues, including the liver [41]. Based on this report, we hypothesized that hexaraphane consumption may delay hepatic senescence by upregulating Sirt1 expression in the livers of aged mice. When examining the mRNA expression levels of mouse p21, encoded by *Cdkn1a*, a key marker of senescence, we observed that *Cdkn1a* mRNA expression levels were increased in both aged groups compared to those in the young control group (Figure 8E). However, this increase was significantly suppressed in the aged hexaraphane group compared to that in the aged control group (Figure 8E). Taken together, these results suggest that administering hexaraphane to aged mice induces Sirt1 upregulation by activating PPARα, thereby establishing a positive feedback loop between Sirt1 and PPARα, while reducing plasma triglyceride levels and attenuating the progression of hepatic senescence.

## 4. Discussion

**Figure 9:**
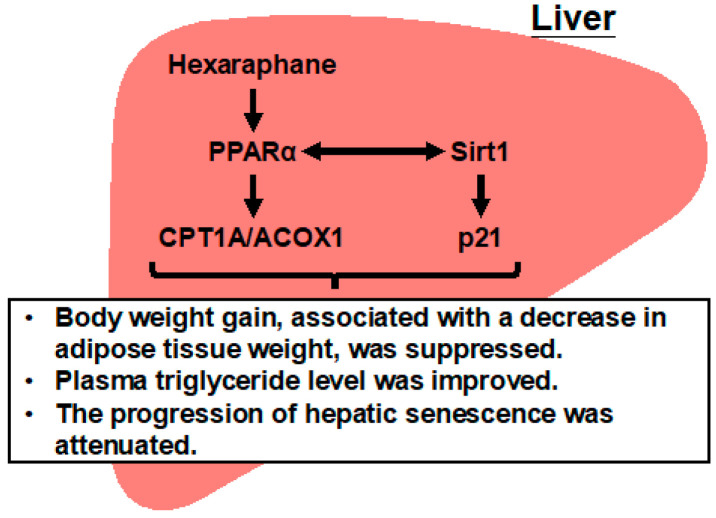
Schematic showing potential regulatory pathways induced by hexaraphane in the liver of aged mice. Hexaraphane increased CPT1A expression and showed a trend toward increased ACOX1 expression by activating PPARα, which was correlated with lower plasma triglyceride levels and decreased body weight gain. Similarly, the increased expression of Sirt1 induced by hexaraphane was associated with lower plasma triglyceride levels. Hexaraphane also increased PPARα expression, suggesting the formation of a positive feedback loop between PPARα and Sirt1. In addition, hexaraphane may delay the progression of hepatic senescence by suppressing age-related increases in p21 expression via upregulation of Sirt1 expression. These observations indicate that hexaraphane-induced upregulation of PPARα target genes may decrease age-related body weight gain by reducing fat weight, which is associated with improved triglyceride metabolism and attenuation of hepatic senescence progression. CPT1A, carnitine palmitoyltransferase 1A; ACOX1, acyl-CoA oxidase 1; PPARα, peroxisome proliferator-activated receptor α; Sirt1, sirtuin 1.

The results of the present study are summarized in Figure 9. Hexaraphane elevated PPRE transcriptional activity and increased the expression levels of CPT1A, a gene targeted by PPARα, in HepG2 cells, indicating its role as a PPARα activator. When orally administered to aged mice, hexaraphane reduced age-related weight gain by decreasing adipose tissue weight. The observed increase in hepatic CPT1A expression, along with a trend toward increased ACOX1 expression, was inversely correlated with plasma triglyceride levels and weight gain. In addition, hexaraphane increased hepatic Sirt1 expression, which was negatively correlated with plasma triglyceride levels. The increase in PPARα expression in the livers of aged mice administered hexaraphane suggests a positive feedback loop between PPARα and Sirt1. The expression of p21, a marker of senescence regulated by Sirt1, was elevated in the livers of aged mice but suppressed by hexaraphane administration. These findings suggest that hexaraphane may alleviate age-related weight gain, elevated plasma triglyceride levels, and progression of hepatic senescence by upregulating PPARα target genes.

The present study demonstrated that the administration of hexaraphane to aged mice led to an increase in PPARα expression levels in the liver. Similarly, hexaraphane increased the mRNA expression levels of human PPARα, encoded by *PPARA*, in HepG2 cells (Supplementary Figure S5). In contrast, a previous study reported that hexaraphane decreased PPARα mRNA expression levels in the same cell line [42]. This discrepancy may be due to the use of different hexaraphane concentrations in these two studies. Other compounds have also shown conflicting results depending on the concentration used. For instance, indoxyl sulfate, a uremic toxin, induces the proliferation of colorectal cancer (CRC) cells at 250 µM (the average serum concentration in patients with end-stage chronic kidney disease) [43,44], whereas 10 mM indoxyl sulfate induces the death of CRC cells [45]. Based on these findings, the concentration of a compound should be determined according to specific research objectives. The present study showed that the increase in CPT1A mRNA expression levels observed in HepG2 cells stimulated with hexaraphane was also reflected in the livers of aged mice administered hexaraphane. Therefore, the concentration of hexaraphane used in the present study on HepG2 cells was considered appropriate for analyzing its effects. 

GW6471 analysis in the present study indicated that PPARα may modulate HO-1 mRNA expression levels at 24 h but not at 3 h following hexaraphane stimulation. We predicted that conjugation with hexaraphane may be responsible for these effects. This is because when isothiocyanates containing hexaraphane are absorbed into cells, they are metabolized mainly via the mercapturic acid pathway, producing various conjugates, including GSH-conjugated forms [19,46]. Based on this prediction, it is plausible that GSH-conjugated hexaraphane activates PPARα, whereas unconjugated hexaraphane activates only Nrf2, or both Nrf2 and PPARα. Consequently, future research should focus on identifying the types of conjugated hexaraphanes and analyzing their effects on Nrf2 and PPARα.

The present study demonstrated that hexaraphane intake significantly upregulated CPT1A expression in the livers of aged mice. In contrast, although there was a trend toward increased ACOX1 expression, this change was not statistically significant. This result may be attributed to the fact that the mice in the present study were not clearly fasted before dissection. This could explain the lack of a significant increase in ACOX1 expression, as the expression of these genes is typically elevated under fasting conditions. In addition, the increased expression of both CPT1A and ACOX1 following hexaraphane intake showed significant negative correlations with reduced plasma triglyceride levels and suppressed weight gain, suggesting that hexaraphane influences the expression of these genes. However, the greater increase in CPT1A expression compared to ACOX1 indicates that the findings of the present study may be more significantly influenced by the mitochondrial FAO pathway than by the peroxisomal FAO pathway.

The current study revealed that hexaraphane intake reduced the expression levels of p21, a gene associated with cellular senescence that is upregulated in the liver due to aging. Suppressing the increased p21 expression in organs, including the liver, extends the natural lifespan, lowers mortality risk in aged mice, and enhances physical function and health status throughout the life cycle [47]. For instance, increased Sirt1 expression in the liver plays a crucial role in maintaining liver health by suppressing arsenic-induced p21 expression and mitigating hepatic senescence [41,48]. Given that increased Sirt1 expression has been associated with longer lifespans and improved healthspans across various species [41,49], it is plausible that the downregulation of p21 expression induced by Sirt1 contributes to these effects. Additionally, as increased Sirt1 expression is induced by PPARα activation, activating PPARα is expected to aid in lifespan extension. Indeed, studies have shown that α-linolenic acid and oleoylethanolamide, which are PPARα ligands, contribute to lifespan extension in *Caenorhabditis elegans* [50,51,52]. Furthermore, in addition to the reduced lifespan observed in PPARα knockout mice [15], it has been noted that liver-specific PPARα-deficient mice develop fatty liver disease as they age, even on a standard diet [53]. These reports suggest that PPARα activation may help extend both lifespan and healthspan in mice. The present study demonstrated that decreased p21 expression was associated with increased PPARα and Sirt1 expression, indicating that hexaraphane intake may prolong both life and healthspan.

In an analysis of aged mice, activation of PPARα by fenofibrate, a typical PPAR ligand, resulted in liver enlargement [54]. In contrast, no hepatic hypertrophy was observed following the intake of hexaraphane in the present study (Supplementary Figure S6). This discrepancy may be due to the superior ability of fenofibrate to elevate PPRE transcriptional activity compared to hexaraphane in the livers of aged mice. Furthermore, although the regenerative capacity of the liver after resection declines with age [55], upregulation of Sirt1 expression mitigates the increase in p21, thereby delaying hepatic senescence and promoting liver regeneration following partial hepatectomy [56]. The present study showed an increase in p21 expression in the livers of aged mice. In contrast, hexaraphane intake resulted in increased Sirt1 expression and decreased p21 expression. These findings suggest that Sirt1, whose expression is upregulated by hexaraphane-induced PPARα activation, may facilitate liver regeneration after partial hepatectomy by reducing p21 expression.

Rats fed a high-carbohydrate, high-fat diet supplemented with Tasmanian wasabi (*Eutrema japonicum*) powder showed a reduction in diet-induced obesity and elevated blood pressure [57]. This study suggests that hexaraphane in Tasmanian wasabi may alleviate these symptoms. However, it remains uncertain whether hexaraphane is involved, and if so, what its mechanism of action may be. The present study demonstrates that hexaraphane affects triglyceride metabolism and body weight gain by increasing CPT1A expression and, to a lesser extent, ACOX1 expression, through PPARα activation. This mechanism could potentially counteract obesity caused by high-carbohydrate and high-fat diets. Furthermore, because blood pressure is elevated in mice with liver-specific knockout of PPARα [58], the activation of PPARα by hexaraphane in the liver may also contribute to the attenuation of obesity-induced blood pressure elevation.

## 5. Conclusions

The present study indicates that hexaraphane may promote triglyceride metabolism and attenuate the progression of hepatic senescence by acting as a PPARα activator. In addition, administration of hexaraphane did not reduce skeletal muscle weight but resulted in reduced adipose tissue weight, which was associated with improved triglyceride metabolism. Based on these findings, continuous long-term consumption of hexaraphane may potentially extend longevity by enhancing physical function and health status without inducing sarcopenia, improving liver function, and attenuating the progression of hepatic senescence. However, as these findings are based on experiments with cultured cells and aged mice, their relevance to the elderly population remains unclear. This limitation should be considered when interpreting the results of the present study. Indeed, age-related changes in PPARα have not been clearly demonstrated in humans, and it is currently considered difficult to determine whether the results of animal experiments are applicable to humans [52]. Despite this, given the observed negative correlation between serum triglyceride levels and cognitive function in elderly individuals [59], along with improvements in certain cognitive functions following the consumption of hexaraphane [18], the findings of the present study may be significant for the elderly. Therefore, future research should determine whether the present findings can be replicated in older populations.

## Figures and Tables

**Table 1:** Forward (Fw) and reverse (Rv) primers were used for the target genes.

Target Genes	GenBank Accession No.	Primers	Location	Length (bp)	Product Length (bp)	Primer Conc. (nM)
Human						
*HMOX1*	NM_002133.3	Fw: CAGTGCCACCAAGTTCAAGC	597–616	20	112	250
		Rv: GTTGAGCAGGAACGCAGTCTT	708–688	21		250
*CPT1A*	NM_001031847.3	Fw: ATGCGCTACTCCCTGAAAGTG	519–539	21	77	250
		Rv: GTGGCACGACTCATCTTGC	595–577	19		250
*PPARA*	NM_005036.6	Fw: ATGGTGGACACGGAAAGCC	337–355	19	124	250
		Rv: CGATGGATTGCGAAATCTCTTG	460–439	22		250
*RPLP0*	NM_001002.4	Fw: CGACCTGGAAGTCCAACTAC	97–116	20	108	250
		Rv: ATCTGCTGCATCTGCTTG	205–188	18		250
Mouse						
*Cpt1a*	NM_013495.2	Fw: CTCCGCCTGAGCCATGAAG	148–166	19	100	250
		Rv: CACCAGTGATGATGCCATTCT	247–227	21		250
*Acox1*	NM_015729.4	Fw: TCGAAGCCAGCGTTACGAG	240–258	19	80	250
		Rv: GGTCTGCGATGCCAAATTCC	319–300	20		250
*Sirt1*	NM_019812.3	Fw: GCTGACGACTTCGACGACG	390–408	19	101	250
		Rv: TCGGTCAACAGGAGGTTGTCT	490–470	21		250
*Ppara*	NM_001113418.1	Fw: AGAGCCCCATCTGTCCTCTC	221–240	20	153	250
		Rv: ACTGGTAGTCTGCAAAACCAAA	373–352	22		250
*Cdkn1a*	NM_001111099.2	Fw: CCTGGTGATGTCCGACCTG	144–162	19	103	250
		Rv: CCATGAGCGCATCGCAATC	246–228	19		250
*Rplp0*	NM_007475.5	Fw: GCTCCAAGCAGATGCAGCA	228–246	19	143	250
		Rv: CCGGATGTGAGGCAGCAG	370–353	18		250

## Data Availability

The original contributions presented in this study are included in this article/Supplementary Materials. Further inquiries can be directed to the corresponding author.

## Supplementary Materials

The following supporting information can be downloaded at: https://www.mdpi.com/article/10.3390/nu17111768/s1, Figure S1: Effect of hexaraphane on the viability of HepG2 cells; Figure S2: Effect of GW6471 on the viability of HepG2 cells; Figure S3: Effects of hexaraphane on the body weights of young control, aged control, and aged hexaraphane groups; Figure S4: Relationship between hepatic Sirt1 mRNA expression levels and Δbody weight in aged mice; Figure S5: Effects of hexaraphane on PPARα expression in HepG2 cells; Figure S6: Effects of hexaraphane on liver weights of aged mice.
