# Supplementary material for: Hexaraphane Affects the Activation of Hepatic PPARα Signaling: Impact on Plasma Triglyceride Levels and Hepatic Senescence with Aging
Source: Nutrients. 2025 May 23;17(11):1768. doi: 10.3390/nu17111768 (PMC12158025; doi:10.3390/nu17111768)
Supplement: Supplementary file 1 [file nutrients-17-01768-s001.zip › nutrients-3551873-supplementary.pdf]

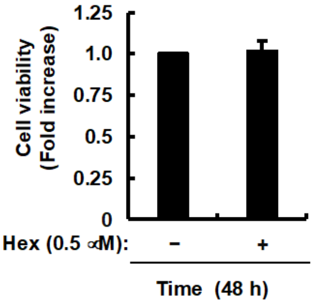

Figure S1. Effect of hexaraphane on the viability of HepG2 cells. Measurement of cell viability. Data are presented as the mean ± SE of three independent experiments. * $p < 0.05$, vs. control solution (DPBS (−)). Hex, hexaraphane.

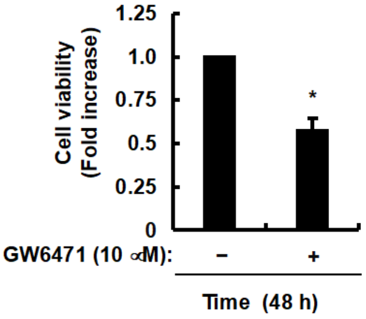

Figure S2. Effect of GW6471 on the viability of HepG2 cells. Measurement of cell viability. Data are presented as the mean ± SE of three independent experiments. * $p < 0.05$, vs. control solution (DMSO).

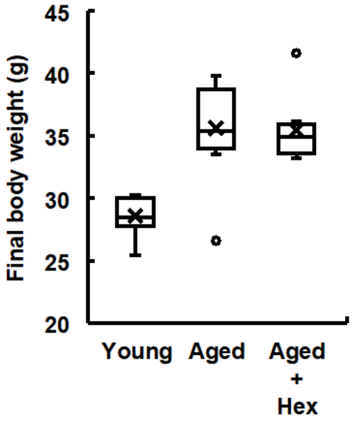

Figure S3. Effects of hexaraphane on the body weights of young control, aged control, and aged hexaraphane groups. The body weights of each group are shown as box plots. Data represent the values, means, medians, and percentiles for each group of mice. Aged, aged control group; Aged + Hex, aged mice administered hexaraphane group; Young, young control group.

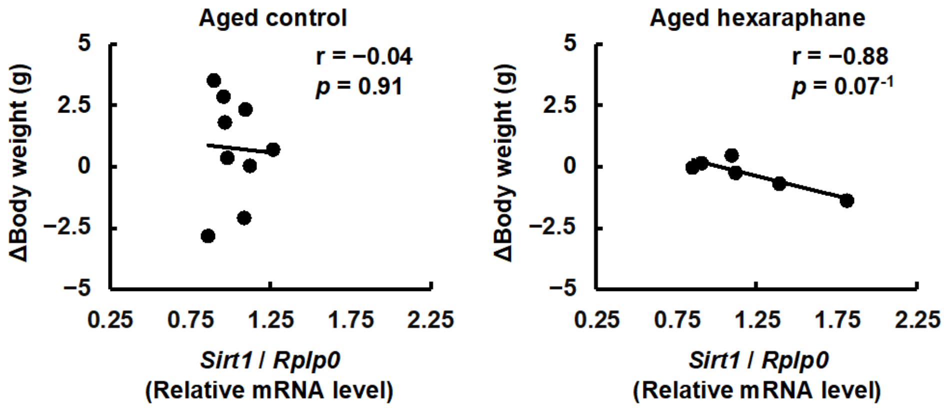

Figure S4. Relationship between hepatic Sirt1 mRNA expression levels and Δbody weight in aged mice. Correlation between hepatic *Sirt1* mRNA expression levels and Δbody weight. The values are presented for each group of mice. Aged control, aged control group; Aged hexaraphane, aged mice administered hexaraphane group.

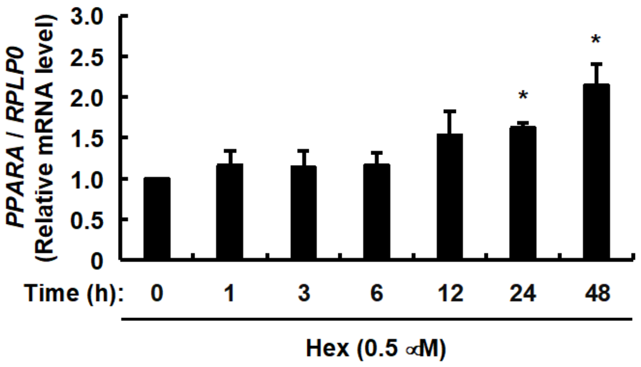

Figure S5. Effects of hexaraphane on PPAR*α* expression in HepG2 cells. Time-dependent of *PPARA* mRNA expression levels. Data are presented as the mean ± SE of four independent experiments. *$p < 0.05$ vs. 0 h. Hex, hexaraphane.

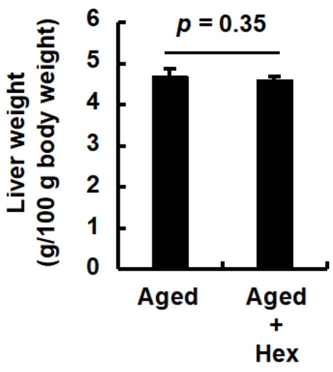

Figure S6. Effects of hexaraphane on liver weights of aged mice. Indicates liver weight relative to body weight. Data are presented as the mean ± SE of four independent experiments. *$p < 0.05$ vs. aged control group. Aged, aged control group; Aged + Hex, aged mice administered hexaraphane group.
